# Supplementary material for: Heat adaptation of phage T7 under an extended genetic code
Source: Virus Evol. 2021 Dec 1;7(2):veab100. doi: 10.1093/ve/veab100 (PMC8923235; doi:10.1093/ve/veab100)
Supplement: veab100_Supp [file veab100_supp.zip › Sequencing_Analysis.zip!/Sequencing_Analysis/A3/evidence/SNP_16.html]

BRESEQ :: Evidence


| Predicted mutation | | | | | | |
| --- | --- | --- | --- | --- | --- | --- |
| evidence | seq id | position | mutation | annotation | gene | description |
| RA | NC\_001604.1 | 37,346 | T→C | intergenic (–/–) | – / – | –/– |

| Read alignment evidence... | | | | | | | | | | | |
| --- | --- | --- | --- | --- | --- | --- | --- | --- | --- | --- | --- |
|  | seq id | position | | ref | new | freq | score (cons/poly) | reads | annotation | genes | product |
| \* | NC\_001604.1 | 37,346 | 0 | T | C | 99.3% | 5472.4 / 0.4 | 1826 | intergenic (–/–) | *–/–* | –/– |
| Reads supporting (aligned to +/- strand):  **ref** base T (6/7);  **new** base C (881/918);  **total** (893/933) | | | | | | | | | | | |
| Fisher's exact test for biased strand distribution *p*-value = 1.00e+00 | | | | | | | | | | | |
| Kolmogorov-Smirnov test that lower quality scores support variant *p*-value = 9.95e-01 | | | | | | | | | | | |

Only 100 of 1826 total aligned reads displayed.  

`AGTTGAGGCTGCGAAGAGCACTCAAAGAGCAATCGATGCGGTATCTGCTAAGTATCAAGAAGACCTTGCCGCGCTGGAAGGGAGCACTGATAGGATTATTTCTGATTTGCGTAGCGACAATAAGCGGTTGCGCGTCAGAGTCAAAACTACCGGAACCTCCGATGGTCAGTGTGGATTCGAGCCTGATGGTCGAGCCGAACTTGACGACCGAGATGCTAAACGTATTCTCGCAGTGACCCAGAAGGGTGACGCATGGATTCGTGCGTTACAGGATACTATTCGTGAACTGCAACGTAAGTAGGAAATCAAGTAAGGAGGCAATGTGT‑CTACTCAATCCAATCGTAATGCGCTCGTAGTGGCGCAACTGAAAGGAGACTTCGTGGCGTTCCTATTCGTCTTATGGAAGGCGCTAAACCTACCGGTGCCCACTAAGTGTCAGATTGACATGGCTAAGGTGCTGGCGAATGGAGACAACAAGAAGTTCATCTTACAGGCTTTCCGTGGTATCGGTAAGTCGTTCATCACATGTGCGTTCGTTGTGTGGTCCTTATGGAGAGACCCTCAGTTGAAGATACTTATCGTATCAGCCTCTAAGG  >  NC_001604.1/37048‑37643`  

                                                                                                                                                                                                                                                                                                          `|`                                                                                                                                                                                                                                                                                                            
`agTTGAGGCTGCGAAGAGCACTCAAAGAGCAATCGATGCGGTATCTGCTAAGTATCAAGAAGACCTTGCCGCGCTGGAAGGGAGCACTGATAGGATTATTTCTGATTTGCGTAGCGACAATAAGCGGTTGCGCGTCAGAGTCAAAACTACCGGAACCTCCGATGGTCAGTGTGGATTCGAGCCTGATGGTCGATCCGAACTTTACGACCGCGATGCTAAACGTATTCTCGCAGTGACCCCGAAGGGTGACGCATGGATTCGTGCGTTACAGTATACTATTCGTGAACTGCAACGTAAGCAg                                                                                                                                                                                                                                                                                                          >  1:335598/1‑301 (MQ=255)`  
      `ggCTGCGGACAGCACTCAACGAGCAATCGATGCGGTATCTGCTAAGTATCAAGAAGACCTTGCCGCGCTGGAAGGGAGCACTGATAGGATTATTTCTGATTTGCGTAGCGACAATAAGCGGTTGCGCGTCAGAGTCAAAACTACCGGAACCTCCGATGGTCAGTGTGGATTCGAGCCTGATGGTCGAGCCGAACTTGACGACCGAGATGCTAAACGTATTCTCGCAGTGACCCAGAAGGGTGACGCATGGATTCGTGCGTTACAGGATACTATTCGTGAACTGCAACGTAAGCAGGAAATc                                                                                                                                                                                                                                                                                                    <  1:92087/301‑1 (MQ=255)`  
                        `aaGAGCAATCGATGCGGTATCTGCTAAGTATCAAGAAGACCTTGCCGCGCTGGAAGGGAGCACTGATAGGATTATTTCTGATTTGCGTAGCGACAATAAGCGGTTGCGCGTAAGAGTCAAAACTACCGGAACCTCCGATGGTCAGTGTGGATTCGAGCCTGATGGTCGAGCCGAACTTGACGACCGAGATGCTAAACGTATTCTCGCAGTGACCCAGAAGGGTGACGCATGGATTCGTGCGTTACAGGATACTATTCGTGAAATGCAACGTAAGAAGGAAATCAAGTAAGGAGGCAAtgtt                                                                                                                                                                                                                                                                                  >  1:175909/1‑300 (MQ=255)`  
                        `aaGAGCAATCGAGGCGGTATCTGCTAAGTATCAAGAAGACCTTGCCGCGCTGGAAGGGAGCACTGATAGGATTATTTCTGATTTGCGTAGCGACAATAAGCGGTTGCGCGTCAGAGTCAAAACTACCGGAACCTCCGATGGTCAGTGTGGATTCGAGCCTGATGGTCGAGCCGAACTTGACGACCGAGATGCTAAACGTATTCTCGCAGTGACCCAGAAGGGTGACGCATGGATTCGTGCGTTACAGGATACTATTCGTGAACTGCAACGTAAGCAGGAAATCAAGTAAGGAGGCAAtgtg                                                                                                                                                                                                                                                                                  <  1:313972/301‑1 (MQ=255)`  
                           `ggCAATCGATGCGGTATCTGCTAAGTATCAAGAAGACCTTGCCGCGCGGGAAGGGAGCACTGATAGGATTATTACTGATTTGCGTAGCGACAATAAGCGGTTGCGCGTCAGAGTCAAAACTACCGGAACCTCCGATGGTCAGTGTGGATTCGAGCCTGATGGTCGAGCCGAACTTGACGACCGAGATGCTAAACGTATTCTCGCAGTGACCCAGAAGGGTGACGCATGGATTCGTGCGTTACAGGATACTATTCGTGAACTGCAACGTAAGCAGGAAATCAAGTAAGGAGGCAATGTGT‑Ct                                                                                                                                                                                                                                                                              <  1:321264/300‑1 (MQ=255)`  
                              `gtataagagacagtCTGCTAAGTATCAAGAAGACCTTGCCGCGCTGGAAGGGAGCACTGATAGGATTATTTCTGATTTGCGTAGCGACAATAAGCGGTTGCGCGTCAGAGTCAAAACTACCGGAACCTCCGATGGTCAGTGTGGATTCGAGCCTGATGGTCGAGCCGAACTTGACGACCGAGATGCTAAACGTATTCTCGCAGTGACCCAGAAGGGTGACGCATGGATTCGTGCGTTACAGGATACTATTCGTGAACTGCAACGTAAGCAGGAAATCAAGTAAGGAGGCAATGTGT‑CTACt                                                                                                                                                                                                                                                                           <  1:199803/288‑1 (MQ=255)`  
                              `aaagagacaggTATCTGCTAAGTTTCAAGAAGACCTTGCCGCGCTGG‑AGGGAGCACTGATAGGATTATTTCTGATTTGCGTAGCGACAATAAGCGGTTGCGCGTCAGAGTCAAAACTACCGGAACCTCCGATGGTCAGTGTGGATTCGAGCCTGATGGTCGAGCCGAACTTGACGACCGAGATGCTAAACGTATTCTCGCAGTGACCCAGAAGGGTGACGCATGGATTCGTGCGTTACAGGATACTATTCGTGAACTGCAACGTAAGCAGGAAATCAAGTAAGGAGGCAATGTGT‑CTACTc                                                                                                                                                                                                                                                                          <  1:184253/292‑1 (MQ=255)`  
                              `aaTCGATGCGGTATCTGCTAAGTATCAAGAAGACCTTGTCGCGCTGGAAGGGAGCACTGATAGGATTATTTCTGATTTGCGTAGCGAAAATAAGCGTTTGCGCGTCAGAGTCAAAACTACCGGAACCTCCGATGGTCAGTGTGGATTCGAGCCTGATGGTCGAGCCGAACTTGACGACCGAGATGCTAAACGTATTCTCGCAGTGACCCAGAAGGGTGACGCATGGATTCGTGCGTTACAGGATACTATTCGTGAACTGCAACGTAAGCAGGAAATCAAGTAAGGAGGCAATGTGT‑CTACt                                                                                                                                                                                                                                                                           <  1:140944/301‑1 (MQ=255)`  
                               `aTCGATGCGGTATCTGCTAAGTATCAAGAAGACCTTGCCGCGCTGGAAGGGAGCACTGATAGGATTATTTCTGATTTGCGTAGCGACAATAAGCGGTTGCGCGTCAGAGTCAAAACTACCGGAACCTCCGATGGTCAGTGTGGATTCGAGCCTGATGGTCGAGCCGAACTTGACGACCGAGATGCTAAACGTATTCTCGCAGTGACCCAGAAGGGTGACGCATGGATTCGTGCGTTACAGGATACTATTCGTGAACTGCAACGTAAGCAGGAAATCAAGTAAGGAGGCAATGTGT‑CTACTc                                                                                                                                                                                                                                                                          <  1:104797/301‑1 (MQ=255)`  
                               `aTCGATGCGGTATCTGCTAAGTATCAAGAAGACCTTGCCGCGCTGGAAGGGAGCACTGATAGGATTATTTCTGATTTGCGTAGCGACAATAAGCGGTTGCGCGTCAGAGTCAAAACTACCGGAACCTCCGATGGTCAGTGTGGATTCGAGCCTGATGGTCGAGCCGAACTTGACGACCGAGATGCTAAACGTATTCTCGCAGTGACCCAGAAGGGTGACGCATGGATTCGTGCGTTACAGGATACTATTCGTGAACTGCAACGTAAGCAGGAAATCAAGTAAGGAGGCAATGTGT‑CTACTc                                                                                                                                                                                                                                                                          <  1:295856/301‑1 (MQ=255)`  
                               `aTCGATGCGGTATCTGCTAAGTATCAAGAAGACCTTGCCGCGCTGGAAGGGAGCACTGATAGGATTATTTCTGATTTGCGTAGCGACAATAAGCGGTTGCGCGTCAGAGTCAAAACTACCGGAACCTCCGATGGTCAGTGTGGATTCGAGCCTGATGGTCGAGCCGAACTTGACGACCGAGATGCTAAACGTATTCTCGCAGTGACCCAGAAGGGTGACGCATGGATTCGTGCGTTACAGGATACTATTCGTGAACTGCAACGTAAGCAGGAAATCAAGTAAGGAGGCAATGTGT‑CTACTc                                                                                                                                                                                                                                                                          >  1:111744/1‑301 (MQ=255)`  
                               `aTCGATGCGGTATCTGCTAAGTATCAAGAAGACCTTGCCGCGCTGGAAGGGAGCACTGATAGGATTATTTCTGATTTGCGTAGCGACAATAAGCGGTCGCGCGTCAGAGTCAAAACTACCGGAACCTCCGATGGTCAGTGTGGATTCGAGCATGATGGTCGAGCCGAACTTGACGACCGAGATGCTAAACGTATTCTCGCAGTGACCCAGAAGGGTGACGCATGGATTCGTGCGTTACAGGATACTATTCGTGAACTGCAACGTAAGCAGGAAATCAAGTAAGGAGGCAATGTGT‑CTACTc                                                                                                                                                                                                                                                                          <  1:32673/301‑1 (MQ=255)`  
                               `aTCGATGCGGTATCTGCTAAGTATCAAGAAGACCTTGCCGCGCTGGAAGGGAGCAATGATAGGATTATTTCTGATTTGCGTAGCGACAATAAGCGGTTGCGCGTCAGAGTCAAAACTACCGGAACCTCCGATGGTCAGTGTGGATTCGAGCCTGATGGTCGAGCCGAACTTGACTACCGAGATGCTAAACGTATTCTCGCAGTGACCCAGAAGGGTGCCGCATGGATTCGTGCGTTACAGGATACTATTCGTGAACTGCAACGTAAGCAGGAAATCAAGTAaggctgtcccttatacacat                                                                                                                                                                                                                                                                           >  1:81047/1‑284 (MQ=255)`  
                                   `aTGCGGTATCTGCTAAGTATCAAGAAGACCTTGCCGCGCTGGAAGGGAGCACTGATAGGATTATTTCTGATTTGCGTAGCGACAATAAGCGGTTGCGCGTCAGAGTCAAAACTACCGGAACCTCCGATGGTCAGTGTGGATTCGAGCCTGATGGTCGAGCCGAACTTGACGACCGAGATGCTAAACGTATTCTCGAAGTGACCCAGACGGGTGACGCCTGGCTTCGTGCGTTACAGGATACTATTCGTGAACTGCAACGTACGCAGGAACTCAAGTAAGGAGGCAATGTGT‑CTACTaaatt                                                                                                                                                                                                                                                                      >  1:30765/1‑300 (MQ=255)`  
                                     `gCGGTATCTGCTAAGTATCAAGAAGACCTTGCCGCGCTGGAAGGGAGCACTGATAGGATTATTTCTGATTTGCGTAGCGACAATAAGCGGTTGCGCGTCAGAGTCAAAACTACCGGAACCTCCGATGGTCAGTGTGGATTCGAGCCTGATGGTCGAGCCGAACTTGACGACCGAGATGATAAACGTATTCTCGCAGTGACCCAGAAGGGTGACGCATGGATTCGTGCGTTACAGGATACTATTCGTGAACTGCAACGTAAGCAGGAAATCAAGTAAGGAGGCAATGTGT‑CTACTcaatcca                                                                                                                                                                                                                                                                    >  1:28999/1‑301 (MQ=255)`  
                                         `tATCTGCTAAGTATCAAGAAGACCTTGCCGCGCTGGAAGGGAGCACTGATAGGATTATTTCTGATTTGCGTAGCGACAATAAGCGGTTGCGCGTCAGAGTCAAAACTACCGGAACCTCCGATGGTCAGTGTGGATTCGAGCCTGATGGTCGAGCCGAACTTGACGACCGAGATGCTAAACGTATTCTCGCAGTGACCCAGAAGGGTGACGCATGGATTCGTGCGTTACAGGATACTATTCGTGAACTGCAACGTAAGCAGGAAATCAAGTAAGGAGGCAATGTGT‑CTACTCAATCCAATCg                                                                                                                                                                                                                                                                <  1:278922/301‑1 (MQ=255)`  
                                              `gCTAAGTATCAAGAAGACCTTGCCGCGCTGGAAGGGAGCACTGATAGGATTATTTCTGATTTGCGTAGCGACAATAAGCGGTTGCGCGTCAGAGTCAAAACTACCGGAACCTCCGATGGTCAGTGTGTATTCGAGCCTGATGGTCGAGCCGAACTTGACGACCGAGATGCTAAACGTATTCTCGCAGTGACCCAGAAGGGTGACGCATGGATTCGTGCGTTACAGGATACTATTCGTGAACTGCAACGTAAGCAGGAAATCAAGTAAGGAGGCAATGTGT‑CTACTCAATCCAATCGTAATg                                                                                                                                                                                                                                                           >  1:269573/1‑301 (MQ=255)`  
                                              `gCTAAGTATCAAGAAGACCTTGCCGCGCTGGAAGGGAGCACTGATAGGATTATTTCTGATTTGCGTAGCGACAATAAGCGGTTGCGCGTCAGAGTCAAAACTACCGGAACCTCCGATGGTCAGTGTGGATTCGAGCCTGATGGTCGAGCCGAACTTGACGACCGAGATGCTAAACGTATTCTCGCAGTGACCCAGAAGGGTGACGCATGGATTCGTGCGTTACAGGATACTATTCGTGAACTGCAACGTAAGCAGTAAATCAAGTAAGGAGGCAATGTGT‑CTACTCAATCCAATCGTAATg                                                                                                                                                                                                                                                           <  1:309736/301‑1 (MQ=255)`  
                                              `gCTAAGTATCAAGAAGACCTTGCCGCGCTGGAAGGGAGCACTGATAGGATTATTTCTGATTTGCGTAGCGACAATAAGCGGTTGCGCGTCAGAGTCAAAACTACCGGAACCTCCGATGGTCAGTGTGGATTCGAGCCTGATGGTCGAGCCGAACTTGACGACCGAGATGCTAAACGTATTCTCGCAGTGACCCAGAAGGGTGACGCATGGATTCGTGCGTTACAGGATACTATTCGTGAACTGCAACGTAAGCAGGAAATCAAGTAAGGAGGCACTGTGT‑CTACTCAATCCAATCGTAATg                                                                                                                                                                                                                                                           >  1:88812/1‑301 (MQ=255)`  
                                                   `gTATCAAGAAGACCTTGCCGCGCTGGAAGGGAGCACTGATAGGATTATTTCTGATTTGCGTAGCGACAATAAGCGGTTGCGCGTCAGAGTCAAAACTACCGGAACCTCCGATGGTCAGTGTGGATTCGAGCCTGATGGTCGAGCCGATCTTGACGGCCGAGATGCTAAACGTATTCTCGCAGTGACCCAGAAGGGTGACGCATGGATTCGTGCGTTACAGGATACTATTCGTGAACTGCAACGTAAGCAGGAAATCAAGTAAGGAGGCAATGTGT‑CTACTCAATCCAATCGTAATGCGCTc                                                                                                                                                                                                                                                      >  1:129231/1‑301 (MQ=255)`  
                                                   `gTATCAAGAAGACCTTGCCGCGCTGGAAGGGAGCACTGATAGGATTATTTCTGATTTGCGTAGCGACAATAAGAGGTTGCGCGTCAGAGTCAAAACTACCGGAACCTCCGATGGTCAGTGTGGATTCGAGCCTGATGGTCGAGACGAACTTGACGAGCGAGATGCTAAACGTATTCTCGCAGGGAGCCAGAAGGGTGAGGCATGGATTCGTGCGTAACGGGATACTATTGGGGAACGGCAACGTAAGCAGGAAATCAAGTAAGAAGGCAATGTGT‑CTAGTAAAACCAAGGGTAATGAGCtg                                                                                                                                                                                                                                                      >  1:34267/1‑300 (MQ=255)`  
                                                       `cAAGAAGACCTTGCCGCGCTGGAAGGGAGCACTGATAGGATTATTTCTGATTTGCGTAGCGACAATAAGCGGTTGCGCGTCAGAGTCAAAACTACCGGAACCTCCGATGGTTAGTGTGGATTCGAGCCTGATGGTCGAGCCGAACTTGACGACCGAGATGCTAAACGTATTCTCGCAGTGACCCAGAAGGGTGACGCATGGATTCGTGCGTTACAGGATACTATTCGTGAACTGCAACGTAAGCAGGAAATCAAGTAAGGAGGCAATGTGT‑CTACTCAATCCAATCGTAATGCGCTCGTAg                                                                                                                                                                                                                                                  >  1:73940/1‑301 (MQ=255)`  
                                                       `cAAGAAGACCTTGCCGCGCTGGAAGGGAGCACTGATAGGATTATTTCTGATTTGCGTAGCGACAATAAGCGGTTGCGCGTCAGAGTCAAAACTACCGGAACCTCCGATAGTCAGTGTGGATTCGAGCCTGATGGTCGAGCCGAACTTGACGACCGAGATGCTAAACGTATTCTCGCAGTGCCCCAGAAGGGTGACGCATGGATTCGTGCGTTACAGGATACTATTCGTGAACTGCAACGTAAGCAGGCAATCAAGTAAGGATGCAATGTGT‑CTACTCAATCCAATCGTAATGCGCTCGTAg                                                                                                                                                                                                                                                  >  1:264682/1‑301 (MQ=255)`  
                                                       `cAAGAAGACCTTGCCGCGCTGGAAGGGAGAACTGATAGGATTATTTCTGAGCTGCGTAGCGACAATAAGCTGTTGCGCGTCAGAGTTAAAACTACCGTAACCTCCGATGGTCAGTGTGGATTCGAGCCTGATGGTCGAGCCGAACTTGACGACCGAGATGCTAACCGTATTCTCGCAGTGACACAGAAGGGTGACGCATGGATTCGTGCGTTACAGGATACTATTCGTGAACTGCAACGTAAGCAGGAAATCAAGTAAGGAGGCAATGTGT‑CTACTCAATCCAATCGTAATGCGCTCGTAg                                                                                                                                                                                                                                                  <  1:18893/301‑1 (MQ=255)`  
                                                         `agaagaCCTTGCCTCGCTGGAAGGTAGCACTGATAGGATTATTTCTGATTTGCGTAGCGACAATAAGCGGTTGCGCGTCAGAGTCAAAACTACCGGAACCTCCGATGGTCAGTGTGGATTCGAGCCTGATGGTCGAGCCGAACTTGACGACCGAGATGCTAAACGTATTCTCGCAGTGACCCAGAAGGGTGACGCATGGATTCGTGCGTTACAGGATACTATTCGTGAACTGCAACGTAAGCAGGAAATCAAGTAAGGAGGCAATGTGT‑CTACTcctgtatcttatacacatctccgagcc                                                                                                                                                                                                                                                >  1:178676/1‑275 (MQ=255)`  
                                                            `agaCCTTGCCGCGCTGGAAGGGAGCACTGATAGGATTATTTCTGTTTTGTGTAGCGACAATAAGCGGTTGCGCGTCAGAGTCAAAACTACCGGAACCTCCGATGGTCAGTGTGGATTCGAGCCTGATGGTCGAGCCGAACTTGACGACCGAGATGCTAAACGTATTCTCGCAGTGACCCAGAAGGGTGTCGCATGGATTCGTGCGTTACAGGATACTATTCGTGAACTGCAACGTAAGCAGGAAATTAAGTAAGGAGGCAATGTGT‑CTACTCAATCCAATCGTACTGCGCTCGTAGTGgcc                                                                                                                                                                                                                                             >  1:236706/1‑300 (MQ=255)`  
                                                            `agaCCTTGCCGCGCTGGAAGGGAGCACTGATAGGATTATTTCTGATTTGCGTAGCGACAATAAGCGGTTGCGCGTCAGAGTCAAAACTACCGGAACCTCCGATGGTCAGTGTGGATTCGAGCCTGATGGTCGAGCCGAACTTGACGACCGAGTTGCTAAACGTATTCTCGCAGTGACCCAGAAGGGTGACGCATGGATTCGTGCGTTACAGGATACTATTCGTGAACTGCAACGTAAGCAGGAAATCAAGTAAGGAGGCAATGTGT‑CTACTCAATCCAATCGTAATGCGCTCGTAGTGgcg                                                                                                                                                                                                                                             >  1:97215/1‑301 (MQ=255)`  
                                                                       `cggagatgtgtagaagagacaGGAGTATTTCTGATTTGCGTAGCGACAATAAGCGGTTGCGCGTCAGAGTCAAAACGACCGGAACCTCCGATGGTCAGTGTGGATTCGAGCCTGATGGTCGAGCCGAACTTGACGACCGAGATGCTAAACGTATTCTCGCAGTGACCCAGAAGGGTGACGCATGGATTCGTGCGTTACAGGATACTATTCGTGAACTGCAACGTAAGCAGGAAATCAAGTAAGGAGGCAATGTGT‑CTACTCAATCCAATCGTAATGCGCTCGTAGTGGCGCAACTGAAAgg                                                                                                                                                                                                                                  <  1:25414/281‑1 (MQ=255)`  
                                                                       `cgcTGGAAGGGAGCACTGATAGGATTATTTCTGATTTGCGTAGCGACAATAAGCGGTTGCGCGTCAGAGTCAAAACTACCGGAACCTCCGATGGTCAGTGTGGATTCGAGCCTGATGGTCGAGCCGAACTTGACGACCGAGATGCTAAACGTATTCTCGCAGTGACCCAGAAGGGTGACGCATGGATTCGTGCGTTACAGGATACTATTCGTGAACTGCAACGTAAGCAGGAAATCAAGTAAGGAGGCAATGTGT‑CTACTAAATCCAATCGTAATGCGCTCGTAGTGGCGCAACTGAAAgg                                                                                                                                                                                                                                  <  1:57223/301‑1 (MQ=255)`  
                                                                         `cTGGAAGGGAGCACTGATAGGATTATTTCTGATTTGCGTAGCGACAATAAGCGGTTGCGCGTCAGAGTCAAAACTACCGGAACCTCCGATGGTCAGTGTGGATTCGAGCCTGATGGTCGAGCCGAACTTGACGACCGAGATGCTAAACGTATTCTCGCAGTGACCCAGAAGGGTGACGCATGGCTTCGTTCGTTACAGGATACTATTCGTGAACTGCAACGTAAGCAGTACCTCATGTAAGGAGGCAATGTGT‑CTACTCAATTCAATCGTAATGCGCTCGTAGTTGCGCAACTGACAGgag                                                                                                                                                                                                                                >  1:283220/1‑301 (MQ=255)`  
                                                                           `tgAAGGGAGCACTGATAGGATGATTTCTGATTTGCGGAGCGACAATAAGCGGTTGCGCGTCAGAGTCAAAACTACCGGAACCTCCGATGGTCAGTGTGGATTTGAGCCTGATGGTCGAGCCGAACTTGACGACCGAGATGCTAAACGTATTCTCGCAGTGACCCAGAAGGGTGACGCATGGATTCGTGCGTTACAGGATACTATTCGTGAACTGCAACGTAAGCAGGAAATCAAGTAAGGAGGCAATGTGT‑CTACTCAATCCAATCGTAATGCGCTCGTAGTGGCGCAACTGAAAGGAGAc                                                                                                                                                                                                                              <  1:100575/300‑1 (MQ=255)`  
                                                                           `ggAAGGGAGCACTGATAGGATTATTTCTGATTTGCGTAGCGACAATAAGCGGTTGCGCGTCAGAGTCAAAACTACCGGAACCTCCGATGGTCAGTGTGGATTCGAGCCTGATGGTCGAGCCGAACTTGACGACCGAGATGCTAAACATATTCTCGCAGTGACCCAGAAGGGTGACGCATGGATTCGTGCGTTACAGGATACTATTCGTGAACTGCAACGTAAGCAGGAAATCAAGTAAGGAGGCAATGTGT‑CTACTCAATCCAATCGTAATGCGCTCGTAGTGGCGCAACTGAAAGGAGAc                                                                                                                                                                                                                              >  1:77159/1‑301 (MQ=255)`  
                                                                                 `gAGCACTGATAGGATTATTTCTGATTTGCGTAGCGACAATAAGCGGTTGCGCGTCAGAGTCAAAACTACCGGAACCTCCGATGGTCAGTGTGGATTCGAGCCTGATGGTCGAGCCGAACTTGACGACCGAGATGCTAAACGTATTCTCGCAGTGACCCAGAAGGGTGACGCATGGATTCGTGCGTTACAGGATACTATTCGTGAACTGCAACGTAAGCAGGAAATCAAGTAAGGAGGCAATGTGT‑CTACTCAATCCAATCGTAATGCGCTctgtctcttatacacatctccgagcccacga                                                                                                                                                                                                                        >  1:107065/1‑271 (MQ=255)`  
                                                                                  `cagaCTGATAGGATTATTTCTGATTTGCGAAGCGACAATAAGCGGTTGCGCGTCAGAGTCAAAACTACCGGAACCTCCGATGTTCAGTGTGGATTCGAGCCTGATGGGCGAGCCGAACTTGACGACCGAGATGCTAAACGTATTCTCGCAGTGACCCAGAAGGGTGACGCATGGATTCGTGCGTTACAGGATACTATTCGTGAACTGCAACGTAAGCAGGAAATCAAGTAAGGAGGCAATGTGT‑CTACTCAATCCAATCGTAATGCGCTCGTAGTGGCGCAACTGAAAGGAGACTTCGTgg                                                                                                                                                                                                                       <  1:299933/298‑1 (MQ=255)`  
                                                                                    `agagacagggATTATTTCTGATTTGCGTAGCGACAATAAGCGGTTGCGCGTCAGAGTCAAAACTACCGGAACCTCCGATGGACAGTGTGGATTCGAGCCTGATGGTCGAGCCGAACTTGACGACCGAGATGCTAAACGTATTCTCGCAGTGACCCAGAAGGGTGACGCATGGATTAGTGCGTTACAGGATACTATTCGTGAACTGCAACGTAAGCAGGAAATCAAGTAAGGAGGCAATGTGT‑CTACTAAATCCAATCGTAATGCGCTCGTAGTGGCGCAACTGAAAGGAGACTTCGTGGCg                                                                                                                                                                                                                     <  1:25021/293‑1 (MQ=255)`  
                                                                                      `cTGATAGGATTATTTCTGATTTGCGTAGCGACAATAAGCGGTTGCGCGTCAGAGTCAAAACTACCGGAACCTCCGATGGTCAGTGTGGATTCGAGCCTGATGGTCGAGCCGAACTTGACGACCGAGATGCTAAACGTATTCTCGCAGTGACCCAGAAGGGTGACGCATGGATTCGTGCGTTACAGGATACTATTCGTGAACTGCAAAGTAAGCAGGAAATCAAGTAAGGAGGCAATGTGT‑CTACTCAATCCAATCGTAATGAGCTCGTAGTGGAGCAACTGAAAGGAGACTTCGTGGCGtt                                                                                                                                                                                                                   >  1:258069/1‑301 (MQ=255)`  
                                                                                             `gATTATTTCTGATTTGCGTAGCGACAATAAGCGGTTGCGCGTCAGAGTCAAAACTACCGGAACCTCCGATGGTCAGTGTGGATTCGAGCCTGATGGTCGAGCCGAACTTGACGACCGAGATGCTAAACGTATTCTCGCAGTGACCCAGAAGGGTGACGCATGGATTCGTGCGTTACAGGATACTATTCGTGAACTGCAACGTAAGCAGGAAATCAAGTAAGGAGGCAATGTGT‑CTACTCAATCCAATCGTAATGCGCTCGTAGTGGCGCAACTGAAAGGAGACTTCGTGGCGTTCCTATTc                                                                                                                                                                                                            <  1:60567/301‑1 (MQ=255)`  
                                                                                                `tattTCTGATTTGCGTAGCGACAATAAGCGGTTGCGCGTCAGAGTCAAAACTACCGGAACCTCCGATGGTCAGTGTGGATTCGAGCCTGATGGTCGAGCAGAACTTGACGACCGAGATGCTAAACGTATTCTCGCAGTGACCCAGAAGGGTGACGCATGGATTCGTGCGTTACAGGATACTATTCGTGAACTGCAACGTAAGCAGGAAATCAAGTAAGGAGGCAATGTGT‑CTACTCAATCCAATCGTAATGCGCTCGTAGTGGCGCAACTGAAAGGAGAcctgtctcttatacacatctcc                                                                                                                                                                                                         >  1:215474/1‑280 (MQ=255)`  
                                                                                                `gagacaggATTTGCGTAGCGACAATAAGCGGTGGCGCGTCAGAGTCAAAACTACCGGAACCTCCGATGGTCAGTGTGGATTCGAGCCTGATGGTCGAGCCGAACTTGACGACCGAGATGCTAAACGTATTCTCGCAGTGACCCAGAAGGGTGACGCATGGATTCGTGCGTTACAGGATACTATTCGTGAACTGCAACGTAAGCAGGAAATCAAGTAATGAGGCAATGTGT‑CTACTCAATCCAATCGTAATGCGCTCGTAGTGGCGCAACTGAAAGGAGACTTCGTGGCGTTCCTATTCGTc                                                                                                                                                                                                         <  1:340167/294‑1 (MQ=255)`  
                                                                                                  `tttCTGATTTGCGTAGCGACAATAAGCGGTTGCGCGTCAGAGTCAAAACTACCGGAACCTCCGATGGTCAGTGTGGATTCGAGCCTGATGGTCGAGCCGAACTTGACGACCGAGATGCTAAACGTATTCTCGCAGTGCCCCAGAAGGGTGACGCATGGATTCGTGCGTTACAGGATACTATTCGTGAACTGCAACGTAAGCAGGAAATCAAGTAAGGAGGCAATGTGT‑CTACTCAATCCAATCGTAATGCGCTCGTAGTGGCGCAACTGAAAGGAGACTTCGTGGCGTTCCTTTTCGTCtt                                                                                                                                                                                                       >  1:145121/1‑301 (MQ=255)`  
                                                                                                       `gATTTGCGTAGCGACAATAAGCGGTTGCGCGTCAGAGTCAAAACTACCGGAACCTCCGATGTTCAGTGTGGATTCGAGCCTGATGGTCGAGCCGAACTTGACGACCGAGATGCTAAACGTATTCTCGCAGTGACCCAGAAGGGTGACGCATGGATTCGTGCGTTACAGGATACTATTCGTGAACTGCAACGTAAGCAGGAAATCAAGTAAGGAGGCAATGTGT‑CTACTCAATCCAATCGTAATGCGCTCGTAGTGGCGCAACTGAAAGGAGACTTCGTGGCGTTCCTATTCGTCTTATGGa                                                                                                                                                                                                  >  1:292707/1‑301 (MQ=255)`  
                                                                                                            `gCGTAGCGACAATAAGTGGTTGCGCGTCAGAGTCAAAACTACCGGAACCTCCGATGGTCAGTGTGGATTCGAGCCTGATGGTCGAGCCGAACTTGACGACCGAGATGCTAAACGTATTCTCGCAGTGACCCAGAAGGGTGACGCATTGATTCGTGCGTTACAGGATACTATTCGTGAACTGCAACGTAAGCAGGAAATCAAGTAAGGAGGCAATGTGT‑CTACTCAATCCAATCGTAATGCGCTCGTAGTGGCGCAACTGAAAGGAGACTTCGTGGctgtctcttatacacatctccgagcc                                                                                                                                                                                             >  1:275382/1‑276 (MQ=255)`  
                                                                                                                  `cGACAATAAGCGGTTGCGCGTCAGAGTCAAAACTACCGGAACCTCCGATGGTCAGTGTGGATTCGAGCCTGATGGTCGAGCCGAACTTGACGACCGAGATGCTAAACGTATTCTCGCAGTGACCCAGAAGGGTGACGCATGGATTCGTGCATTACAGGATACTATTCGTGAACTGCAACGTAAGCAGGAAATCAAGTAAGGAGGCAATGTGT‑CTACTCAATCCAATCGTAATGCGCTCGTAGTGGCGCAACTGAAAGGAGACTTCGTGGCGTTCCTATTCGTCTTATGGAAGGCGCTAAAc                                                                                                                                                                                       <  1:114576/301‑1 (MQ=255)`  
                                                                                                                         `aaGCGGTTGCGCGTCAGAGTCAAAACTACCGGAACCTCCGATGGTCAGTGTGGATTCGAGCCTGATGGTCGAGCCGAACTTGACGACCGAGATGCTAAACGTATTCTCGCAGTGACCCAGAAGGGTGACGCATGGATTCGTGCGTTACAGGATACTATTCGTGAACTGCAACGTAAGCAGGAAAGCAAGTAAGGAGGCAATGGGT‑CTACTCAATCCAATCGTAATGCGCTCGTAGTGGCGCAACTGAAAGGAGACTTCGTGGCGTTCCTATTCGTCTTATGGAAGGCGCTAAACCTACCgg                                                                                                                                                                                <  1:95470/301‑1 (MQ=255)`  
                                                                                                                         `aaGCGGTTGCGCGTCAGAGTCAAAACTACCGGAACCTCCGATGGTCAGTGTGGATTCGAGCCTGATGGTCGAGCCGAACTTGACGACCGAGATGCTAAACGTATTCTCGCAGTGACCCAGAAGGATGACGCATGGATTCGTGCGTTACAGGATACTATTCGTGAACTGCAACGTAAGCAGGAAATCAAGTAAGGAGGCAATGTGT‑CTACTCAATCCAATCGTAATGCGCTCGTAGTGGCGCAACTGAAAGGAGACTTCGTGGCGTTCCTATTCGTCTTATGGAAGGCGCTAAACCTACCgg                                                                                                                                                                                <  1:151165/301‑1 (MQ=255)`  
                                                                                                                              `gTTGCGCGTCAGAGTCAAAACTACCGGAACCTCCGATGGTCAGTGTGGATTCGAGCCTGATGGTCGAGCCGAACTTGACGACCGAGATGCTAAACGTATTCTCGCAGTGACCCAGAAGGGTGACGCATGGATTCGTGCGTTACAGGATACTATTCGTGAACTGCAACGTAAGCAGGAAATCAAGTAAGGAGGCAATGTGT‑CTACTCAATCCAATCGTAATGCGCTCGTAGTGGCGCAACTGAAAGGAGACTTCGTGGCGTTCCTATTCGTCTTATGGAAGGCGCTAAACCTAACggctgac                                                                                                                                                                           >  1:280373/1‑296 (MQ=255)`  
                                                                                                                                      `tCAGAGTCAAAACTACCGGAACCTCCGATGGTCAGTGGGGATTCGAGCCTGATGGTCGAGCCGAACTTGACGACCGAGATGCTAAACGTATGCTCGCAGTGACCCAGAAGGGTGACGCATGGATTCGTGCGTTACAGGATACTATTCGTGAACTGCAACGTAAGCAGGAAATCAAGTAAGGAGGCAATGTGT‑CTACTCAATCCAATCGTAATGCGCTCGTAGTGGCGCAACTGAAAGGAGACTTCGTGGCGTTCCTATTCGTCTTATGGAAGGCGCTAAACCTACCGGTGCCCACTAAgtg                                                                                                                                                                   <  1:338526/301‑1 (MQ=255)`  
                                                                                                                                            `tCAAACCTACCGTAGCCTCCGAGGGTCAGTATGGATCCGAGCCTGCTGGTCAAGCCGAACTCGACGACCGCGATGCTAAACGTATTCTCGCAGTGACCCAGAAGGGTTACGCATGGATGCGTGCGTTACAGGATACTATTCATGAGCTGCAACGAAAGCAAGAAATCAAGTAAGTAGTCAAAGTGT‑CTACTCAATCCAATCGTAATGCGCTCGTAGTGGCGCAACTGAAAGGACACTTCGTGGCGTTCCTATTCGTCTTATGGAAGGCGCTAATCCTACCGGTGCCCACTAAGTGTCAGAt                                                                                                                                                             <  1:196741/301‑1 (MQ=255)`  
                                                                                                                                            `acagaaCTACCGGAACCCCCGATGGGCAGGGTGGATTCGAGCCTGATGGTCGCGCCGAACTTGACGAACGAGATGCTAAACGTAGTCTCGCAGTGACCCAGAAGGGTGACGCATGGATTCGTGCGTTACAGGATACTATTCGTGAACTGCAACGTAAGCAGGAAATCAAGTAAGGAGGCAATGTGT‑CTACTCAATCCAATCGTAATGCGCTCGTAGTGGCGCAACTGAAAGGAGACTTCGTGGCGTTCCTATTCGTCTTATGGAAGGAGCTAAACCTACCGGTGCCCACTAAGTGTCAGAt                                                                                                                                                             <  1:18709/297‑1 (MQ=255)`  
                                                                                                                                                  `cTACCGGAACCTCCGATGGTCAGTGTGGATTCGAGCCTGATGGTCGAGCCGAACTTGACGACCGAGATGCTAAACGTATTCTCGCAGTGACCCAGAAGGGTGACGCATGGATTCGTGCGTTACAGGATACTATTCGTGAACTGCAACGTAAGCAGGAAATCAAGTAAGGAGGCAATGTGT‑CTACTCAATCCAATCGTAATGCGCTCGTAGTGGCGCAACTGAAAGGAGACTTCGTGGCGTTCCTATTCGTCTTATGGAAGGCGCTAAACCTACCGGTGCCTACTAAGTGTCAGATTGACAt                                                                                                                                                       <  1:148773/301‑1 (MQ=255)`  
                                                                                                                                                    `aCCGGAACCTCCGATGGTCAGTGTGGATTCGAGCCTGATGGTCGAGCCGAACTTGACGACCGAGATGCTAAACGTATTCTCGCAGTGACCCAGAATGGTGACGCATGGATTCGTGCGTTACAGGATACTATTCGTGAACTGCAACGTAAGCAGGAAATCAAGTAAGGAGGCAATGTGT‑CTACTCAATCCAATCGTAATGCGCTCGTAGTGGCGCAACTGAAAGGAGACTTCGTGGCGTTCCTATTCGTCTTATGGAAGGCGCTAAACCTAACGGTGCCCACTAAGTGTCAGATTGACATgg                                                                                                                                                     >  1:83826/1‑301 (MQ=255)`  
                                                                                                                                                          `gatgtgtataagagacagggATTCGAGCCTGATGGTCGAGCCGAACTTGACGACCGAGATGCTAAACGTATTCTCGCAGTGACCCAGAAGGGTGACGCATGGATTCGTGCGTTACAGGATACTATTCGTGAACTGCAACGTAAGCAGGAAATCAAGTAAGGAGGCAATGTGT‑CTACTCAATCCAATCGTAATGCGCTCGTAGTGGCGCAACTGAAAGGAGACTTCGTGGCGTTCCTATTCGTCTTATGGAAGGCGCTAAACCTACCGGTGCCAACTAAGTGTCAGATTGACATGGCTAAgg                                                                                                                                               <  1:38329/283‑1 (MQ=255)`  
                                                                                                                                                          `aCCTCCGATGGTCAGTGTGGATTCGAGCCTGATGGTCGAGCCGAACTTGACGACCGAGATGCTAAACGTATTCTCGCAGTGACCCAGAAGGGTGACGCATGGATTCGTGCGTTACAGGATACTATTCGTGAACTGCAACGTAAGCAGGAAATCAAGTAAGGAGGCAATGTGT‑CTACTCAATCCAATCGTAATGCGCTCGTAGTGGCGCAACTGAAAGGAGACTTCGTGGCGTTCCTATTCGTCTTATGGAAGGCGCTAAACCTACCGGTGCCCACTAAGTGTCAGATTGACATGGCTAAgg                                                                                                                                               <  1:137374/301‑1 (MQ=255)`  
                                                                                                                                                          `aCCTCCGATGGTCAGTGTGGATTCGAGCCTGATGGTCAGGCCAAACTTGACGACCGAGATGCTAAACGTATTCTCGCAGTGACCCAGAAGGGTGACGCATGGATTCGTGCGTTACAGGATACTATTCGGGAACTGCAACGTAAGCAGGAAATCAAGTAAGGAGGCAATGTGT‑CTACTCAATCCAATCGTAATGCGCTCGTAGTGGCGCAACTGAAAGGAGACTTCGTGGCGTTCCTATTTGTCTTATGGAAGGCGCTAAACCTACCGGTGCCCACTAAGTGTCAGATTGACATGGTTAAgg                                                                                                                                               <  1:126501/301‑1 (MQ=255)`  
                                                                                                                                                              `ccGATGGTCAGTGTGGATTCGAGCCTGATGGTCGAGCCGAACTTGACGACCGAGATGCTAAACGTATTCTCGCAGTGACCCAGAAGGGTGACGCATGGATTCGTGCGTTACAGGATACTATTCGTGAACTGCAACGTAAGCAGGAAATCAAGTAAGGAGTCAATGTGT‑CTACTCAATCCAATCGTAATGCGCTCGTAGTGGCGCAACTGAAAGGAGACTTCGTGGCGTTCCTATTCGTCTTATGGAAGGCGCTAAACCTACCGGTGCCCACTAAGTGTcctgtcccttatacacatctccg                                                                                                                                           >  1:222151/1‑279 (MQ=255)`  
                                                                                                                                                                  `gtgggctcggagatgtgtataagggacaggAGCCGAACTTGACGACCGAGATGCTAAACGTATTCTCGCAGTGACCCAGAAGGGTGACGCATGGATTCGTGCGTTACAGGATACTATTCGTGAACTGCAACGTAAGCAGGAAATCAAGTAAGGAGGCAATGTGT‑CTACTCAATCCAATCGTAATGCGCTCGTAGTGGCGCAACTGAAAGGAGACTTCGTGGCGTTCCTATTCGTCTTATGGAAGGCGCTAAACCTACCAGTGCCCACTAAGTGTCAGATTGACATGGCTAAGGTGCTGGCg                                                                                                                                       <  1:55085/272‑1 (MQ=255)`  
                                                                                                                                                                   `ggTCAGTGTGGATTCGAGCCTGATGGTCGAGCCGAACTTGACGACCGAGATGCTAAACGTATTCTCGCAGTGACCCAGAAGGGTGACGCATGGATTCGTGCGTTACATGATACTATTCGTGAACTGCAACGT‑AGCAGGAAATCAAGTAAGGAGGCAATGTGT‑CTACTCAATCCAATCGTAATGCGCTCGTAGTGGCGCAACTGAAAGGAGACTTCGTGGCGTTCCTATTCGTCTTATGGAAGGCGCTAAACCTACCGGTGCCCACTAAGTGTCAGATTGACATGGCTAAGGTGCTgtctct                                                                                                                                     >  1:234004/1‑296 (MQ=255)`  
                                                                                                                                                                   `ggTCAGTGTGGATTCGAGCCTGATGGTCGAGCCGAACTTGACGACCGAGATGCTAAACGTATTCTCGCAGTGACCCAGAAGGGTGACGCATGGATTCGTGCGTTACAGGATACTATTCGTGAACTGCAACGTAAGCAGGAAATCAAGTAAGGAGGCAATGTGT‑CTACTCAATCCAATCGTAATGCGCTCGTAGTGGCGCAACTGAAAGGAGACTTCGTGGCGTTCCTATTCGTCTTATGGAAGGCGCTAAACCTACCGGTGCCCACTAAGTGTCAGATTGACATGGCTAAGGTGcctgtct                                                                                                                                      >  1:70975/1‑295 (MQ=255)`  
                                                                                                                                                                        `gtgtGGATTCGAGCCTGATGGTCGAGCCGAACTTGACGACCGAGATGCTAAACGTATTCTCGCAGTGACCCAGAAGGGTGACGCATGGATTCGTGCGTTACAGGATACTATTCGTGAACTGCAACGTAAGCAGGAAATCAAGTAAGGAGGCAATGTGT‑CTACTCAATCCAATCGTAATGCGCTCGTAGTGGCGCAACTGAAAGGATACTTCGTGGCGTTCCTATTCGTCTTATGGAAGGCGCTAAACCTACTGGTGCCCACTAAGTGTCAGAtctgcctcttatacacatccccgagccca                                                                                                                                 >  1:64451/1‑273 (MQ=255)`  
                                                                                                                                                                            `ggATTCGAGCCTGATGGTCTAGCCGAACTTGACGACCGAGATGCTAAACGTATTCTCGCAGTGACCCAGAAGGGTGACGCATGGATTCGTGCGTTACAGGATACTATTCGTGAACTGCAACGTAAGCAGGAAATCAAGTAAGGAGGCAATGTGT‑CTACTCAATCCAATCGTAATGCGCTCGTAGTGGCGCAACTGAAAGGAGACTTCGTGGCGTTCCTATTCGTCTTATGGAAGGCGCTAAACCTACCGGTGCCCACTAAGTGTCAGATTGACATGGCTAAGGTGCTGGCgctgtctctta                                                                                                                             >  1:332650/1‑291 (MQ=255)`  
                                                                                                                                                                             `gATTCGAGCCTGATGGTCGAGCCGAACTTGACGACCGAGATGCTAAACGTATTCTCGCAGTGACCCAGAAGGGTGACGCATGGATTCGTGCGTTACAGGATACTATTCGTGAACTGCAACGTAAGCAGGAAATCAAGTAAGGAGGCAATGTGT‑CTACTCAATCCAATCGTAATGCGCTCGTAGTGGCGCAACTGAAAGGAGACTTCGTGGCGTTCCTATTCGTCTTATGGAAGGCGCTAAACCTACCGGTGCCCACTAAGTGTCAGATTGACATGGCTAAGGTGTTGGCGAATGGAGacaa                                                                                                                            >  1:86556/1‑301 (MQ=255)`  
                                                                                                                                                                                     `ccTGATGGTCGAGCCGAACTTGACGACCGAGATGCTAAACGTATTCTCGCAGTGACCCAGAAGGGTGCCGCATGGATTCGTGCGTTACAGGATACTATTCGTGAACTGCAACGTACGCAGGAAATCAAGTAAGGAGGCAATGTGT‑CTACTCAATCCAATCGTAATGCGCTCGTAGTGGCGCAACTGAAAGGAGCCTTCGTGGCGTTCCTATTCGTCTTATGGAAGGCGCTAAACCTACCGGTGCCCACTAAGTTTCAGATTGACATGGCTAAGGTGCTTGCGAATGGAGACACCAATAAGt                                                                                                                    >  1:173087/1‑301 (MQ=255)`  
                                                                                                                                                                                            `gTCGAGCCGAACTTGACGACCGAGATGCTAAACGTATTCTCGCAGTGACCCAGAAGGGTGACGCATGGATTCGTGCGTTACAGGATACTATTCGTGAACTGCAACATAAGCAGGAAATCAAGTAAGGAGGCAATGTGT‑CTACTCAATCCAATCGTAATGCGCTCGTAGTGGCGCAACTGAAAGGAGACTTCGTGGCGTTCCTATTCGTCTTATGGAAGGCGCTAAACCTACCGGTGCCCACTAAGTGTCAGATTGACATGGCTAAGGTGCTGGCGAATGGAGACAACaagctgtctcttat                                                                                                             >  1:303935/1‑290 (MQ=255)`  
                                                                                                                                                                                                `aGCCGAACTTGACGACCGAGATGCTAAACGTATTCTCGCAGTGACCCAGAAGGGTGACGCATGGATTCGTGCGTTACAGGATACTATTCGTGAACTGCAACGTAAGCAGGAAATAAAGTAAGGAGGCAATGTGT‑CTACTCAATCCAATCGTAATGCGCTCGTAGTGGCGCAACTGAAAGGAGACTTCGTGGCGTTCCTATTCGTCTTATGGAAGGCGCTAAACCTACCGGTGCCCACTAAGTGTCAGATTGACATGGCTAAGGTGCTGGCGAATGGAGACAACAAGAAGTTCATcctgtct                                                                                                         >  1:133691/1‑295 (MQ=255)`  
                                                                                                                                                                                                 `gtcggagatgtgtaaaagggacagaaaCGTATTCTCGCAGTGACCCAGAAGGGTGACGCATGTATTCGTGCGTTACAGGATACTATTCGTGAACTGCAACGTAAGCTGGAAATCAAGTAAGGAGGCAATGTGT‑CTACTCAATCCAATCGTAATGCGCTCGTAGTGGCGCAACTGAAAGGAGACTTCGTGGCGTTCCTATTCGTCTTATGGAAGGCGCTAAACCTACCGGTGCCCACTAAGTGTCAGATTGACATGGCTAAGGTGCTGGCGAATGGAGACAACAAGAAGTTCATCTTACAgg                                                                                                        <  1:11701/277‑1 (MQ=255)`  
                                                                                                                                                                                                 `gCCCAACTGGACGACCGAGATGCTAAAAGATGTATAGCAGTAACCCAGAAGGAAGACTTATTGATGCGTGCTTTACAGGATACTATTCGGGAACTGCATCGTAAGCAGGAAATCAAGTAAGGATGCAATGTGT‑CTACGCAATCCAATCGTAATGCGCTCGTGTTGGCGCCACTGAAAGGAGAGTTCGTGGCGTTCCTATTCTTCTTATGGTAGTCGCTAAACCTACCGGTGCCCACTAAGTGTCAGATTTACATGGCTAAGGTGCTGGCGAATGGAGACAACAAGAAGTTCATCTTACAgg                                                                                                        <  1:44693/301‑1 (MQ=255)`  
                                                                                                                                                                                                  `ccGAACTTGACGACCGAGATGCTAAACGTATTCTCGCAGTGACCCAGAAGGGTGACGCATGGATTCGTGCGTTACAGGATACTATTCGTGAACTGCAACGTAAGCAGGAAATCAAGTAAGGAGGCAATGTGT‑CTACTCAATCCAATCGTAATGCGCTCGTAGTGGCGCAACTGAAAGGAGACTTCGTGGCGTTCCTATTCGTCTTATGGAAGGCGCTAAACCTACCGGTGCCCACTAAGTGTCAGATTGACATGGCTAAGGTGCTGGCGAATGGAGACAACAAGAAGTTCATCTTACAGGc                                                                                                       <  1:169/301‑1 (MQ=255)`  
                                                                                                                                                                                                      `ggtgtgctcggagatgtgtataagagacagcGCAGTGACCCAGAAGGGTGACGCATGGATTCGTGCGTTACAGGATACTATTCGTGAACTGCAACGTAAGCAGGAAATCAAGTAAGGAGGCAATGTGT‑CTACTCAACCCAATCGTAATGCGCTCGTAGTGGCGCAACTGAAAGGAGACTTCGTGGCGTTCCTATTCGTCTTATGGAAGGCGCTAAACCTACCGGTGCCCACTAAGTGTCAGATTGACATGGCTAAGGTGCTGGCGAATGGAGACAACAAGAAGTTCATCTTACAGGCTTTc                                                                                                   <  1:218436/271‑1 (MQ=255)`  
                                                                                                                                                                                                         `taacgacCGAGAGACTAAACGTATTCTCGCAGTGACCCAGAAGGGGGACGCATGGATTCGTGCGTTACAGGATACTATTCGTGAACTGCAACGTAAGCAGGAAATCAAGTAAGGAGGCAATGTGT‑CTACTCAATCCAATCGTAATGCGCTCGTAGTGGCGCAACTGAAATGAGACTTCGTGGCGTTCCTATTCGTCTTATGGAAGGCGCTAAACCTACCGGTGCCCACTAAGTGTCAGATTGACATGGCTAAGGTGCTGGCGAATGGAGACAACAAGAAGTTCATCTTACAGGCTTTCCGt                                                                                                <  1:243970/299‑1 (MQ=255)`  
                                                                                                                                                                                                                   `gaTGCTAAACGTATTCTCGCAGTGACCCAGAAGGGTGACGCATGGATTCGTGCGTTACAGGATACTATTCGTGAACTGCAACGTAAGCAGGAAATCAAGTAAGGAGGCAATGTGT‑CTACTCAATCCAATCGTAATGCGCTCGTAGTGGCGCAACTGAAAGGAGACTTCGTGGCGTTCCTATTCGTCTTATGGAAGGCGCTAAACCTACCGGTGCCCACTAAGTGTCAGATTGACATGGCTAAGGTGCTGGCGAATGAAGACAACAAGAAGTTCATCTTACAGGCTTTcctgtctcttatac                                                                                      >  1:181953/1‑289 (MQ=255)`  
                                                                                                                                                                                                                     `tGCTAAACGTATTCTCGCAGTGACCCAGAAGGGTGACGCATGGATTCGTGCGTTACAGGATACTATTCGTGAACTGCAACGTAAGCAGGAAATCAAGTAAGGAGGCAATGTGT‑CTACTCAATCCAATCGTAATGCGCTCGTAGTGGCGCAACTGAAAGGAGACTTCGTGGCGTTCCTATTCGTCTTATGGAAGGCGCTAAACCTACCGGTGCCCACTAAGTGTCAGATTGACATGGCTAAGGTGCTGGCGAATGGAGACAACAAGAAGTTCATCTTACAGGCTTTCCGTGGTATCGGTAAg                                                                                    >  1:247232/1‑301 (MQ=255)`  
                                                                                                                                                                                                                           `aCGTATTCTCGCAGTGACCCAGAAGGGTGACGCATGGATTCGTGCGTTACAGGATACTATTCGTGAACTGCAACGTAAGCAGGAAATCAAGTAAGGAGGCAATGTGT‑CTACTCAATCCAATCGTAATGCGCTCGTAGTGGCGCAACTGAAAGGAGACTTCGTGGCGTTCCTATTCGTCTTATGGAAGGCGCTAAACCTACCGGTGCCCACTCAGTGTCAGATTGACATGGCTAAGGTGCTTGAGAATGGAGACAACCAGAAGTTCATCTTTCAGGCTTTCCGTGGTACCGGTAAGTCGtta                                                                              >  1:48277/1‑300 (MQ=255)`  
                                                                                                                                                                                                                                `ttttcGCATTGAACCAGAAAGATGACGTATGGATTCTTGCGTTAAAGGTTATTATTCGTGAACGTCAACGTAAGCAGGAAATCAAGTAAGGATGCAATGTGT‑CTACTCAATCCAATCGTAATGCGCTCGTAGTGGCGCAACTGACAGGAGACTTCGTGGCGTTCATATTCTTCTTATGGAAGGCGCTAAACCTACCGGTGCCCACTAAGTGTCAGATTGACATGGCTAAGGTGCTGGCGAATGGAGACAATAAGAAGTTAATATTACAGGCTTTCCGTGGTATCGGTAAGTCGTTCATcac                                                                         <  1:121683/298‑1 (MQ=255)`  
                                                                                                                                                                                                                                       `aGTGACCCAGAAGGTGGACCAATGGATTCGTGCGTTACAGGATACTATTCGTGAACTGCAACGTAAGCAGGAAATCAAGTAAGGAGGCAATGTGT‑CTACTCAATCCAATCGTAATGCGCTCGTAGTGGCGCAACTGAAAGGAGACTTCGTGGCGTTCCTATTCGTCTTATTGAAGGCGCTAAACCTACCGGTGCCCACTAAGTGTCAGATTGACATGGCTAAGGTGCTGGCGAATGGAGACAACAAGAAGTTCATCTTACAGGCTTTCCGTGGTATCGGTAAGTCGTTCATCACATGTGcg                                                                  <  1:157246/301‑1 (MQ=255)`  
                                                                                                                                                                                                                                          `gACCCAGAAGGGTGACGCATGGATTCGTGCGTTACAGGATACTATTCGTGAACTGCAACGTAAGCAGGAAATCAAGTAAGGAGGCAATGTGT‑CTACTCAATCCAATCGTAATGCGCTCGTAGTGGCGCAACTGAAAGGAGACTTCGTGGCGTTCCTATTCGTCTTATGGAAGGCGCTAAACCTACCGGTGCCCACTAAGTGTCAGATTGACATGGCTAAGGTGCTGGCGAATGGAGACAACAAGAAGTTCATCTTACAGGCTTTCCGTGGTATCGGTAAGTCGTTCATCACATGTGcgttc                                                               >  1:260575/1‑301 (MQ=255)`  
                                                                                                                                                                                                                                                `gAAGGGTGACGCATGGATTCGTGCGTTACAGGATACTATTCGTGAACTGCAACGTAAGCAGGAAATCAAGTAAGGAGGCAATGTGT‑CTACTCAATCCAATCGTAATGCGCTCGTAGTGGCGCAACTGAAAGGAGACTTCGTGGCGTTCCTATTCGTCTTATGGAAGGCGCTAAACCTACCGGTGCCCACTAAGTGTCAGATTGACATGGCTAAGGTGCTGGCGAATGGAGACAACAAGAAGTTCATCTTACAGGCTTTCCGTGGTATCGGTAAGTCGTTCATCACATGTGCGTTCGTtgtg                                                         <  1:241069/301‑1 (MQ=255)`  
                                                                                                                                                                                                                                                  `aGGGGGATGCATGGATTCGTTCGTTACAGGATACTATTCGTGAACTGCAACGTAAGCAGGAAATCAAGTAAGGAGGCAATGTGT‑CTACTCAATCCAATCGTAATGCGCTCGTAGTGGCGCAACTGAAAGGAGACTTCGTGGCGTTCCTATTCGTCTTATGGAAGGCGCTAAACCTACCGGTGCCCACTAAGTGTCAGATTGACATGGCTAAGGTGCTGGCGAATGGAGACAACAAGAAGTTCATCTTACAGGCTTTCCGTGGTATCGGTAAGTCGTTCATCACATGTGCGTTCGTtgtgtg                                                       <  1:312110/301‑1 (MQ=255)`  
                                                                                                                                                                                                                                                   `gggTGACGCATGGATTCGTGCGTTACAGGATACTATTCGTGAACTGCAACGTAAGCAGGAAATCAAGTAAGGAGGCAATGTGT‑CTACTCAATCCAATCGTAATGCGCTCGTAGTGGCGCAACTGAAAGGAGACTTCGTGGCGTTCCTATTCGTCTTATGGAAGGCGCTAAACCTACCGGTGCCCACTAAGTGTCAGATTGACATGGCTAAGGTGCTGGCGAATGGAGACAACAAGAAGTTCATCTTACAGGCTTTCCGTGGTATCGGTAAGTCGTTCATCACATGTGCGTTCGTTGTGTgg                                                      <  1:330265/301‑1 (MQ=255)`  
                                                                                                                                                                                                                                                    `ggTGACGCATGGATTCGTGCGTTACAGGATACTATTCGTGAACTGCAACGTAAGCAGGAAATCAAGTAAGGAGGCAATGTGT‑CTACTCAATCCAATCGTAATGCGCTCGTAGTGGCGCAACTGAAAGGAGACTTCGTGGCGTTCCTATTCGTCTTATGGAAGGCGCTAAACCTACCGGTGCCCACTAAGTGTCAGATTGACATGGCTAAGGTGCTGGCGAATGGAGACAACAAGAAGTTCATCTTACAGGCTTTCCGTGGTATCGGTAAGTCGTTCATCACATGTTCGTTCGTTGTGTGGt                                                     >  1:51740/1‑301 (MQ=255)`  
                                                                                                                                                                                                                                                           `cATGGATTCGTGCGTTACAGGATACTATTCGTGAACTGCAACGTAAGCAGGAAATCAAGTAAGGAGGCAATGTGT‑CTACTCAATCCAATCGTAATGCGCTCGTAGTGGCGCAACTGAAAGGAGACTTCGTGGCGTTCCTATTCGTCTTATGGAAGGCGCTAAACCTACCGGTGCCCACTAAGTGTCAGATTGACATGGCTAAGGTGCTGGCGAATGGAGACAACAAGAAGTTCATCTTACAGGCTTTCCGTGGTATCGGTAAGTCGTTCATCACATGTGCGTTCGTTGTGTGGTCCTTATg                                              <  1:125062/301‑1 (MQ=255)`  
                                                                                                                                                                                                                                                            `gtgtataagagacagaCAGGATACTATTCGTGAACTGCAACGTAAGCAGGAAATCAAGTAAGGAGGCAATGTGT‑CTACTCAATCCAATCGTAATGCGCTCGTAGTGGCGCAACTGAAAGGAGACTTCGTGGCGTTCCTATTCGTCTTATGGAAGGCGCTAAACCTACCGGTGCCCACTAAGTGTCAGATTGACATGGCTAAGGTGCTGGCGAATGGAGACAACAAGAAGTTCATCTTACAGGCTTTCCGTGGTATCGGTAAGTCGTTCATCACATGTGCGTTCGTTGTGTGGTCCTTATgg                                             <  1:192886/286‑1 (MQ=255)`  
                                                                                                                                                                                                                                                              `gtataagagacagaCAGGATACTAGTCGTGAACTGCAACGTAAGCAGGAAATCAAGTAAGGAGGCAATGTGT‑CTACTCAATCCAATCGTAATGCGCTCGTAGTGGCGCAACTGAAAGGAGACTTCGTGGCGTTCCTATTCGTCTTATGGAAGGCGCTAAACCTACCGGTGCCCACTAAGTGTCAGATTGACATGGCTAAGGTGCTGGCGAATGGAGACAACAAGAAGTTCATCTTACAGGCTTTCCGTGGTATCGGTAAGTCGTTCATCACATGTGCGTTCGTTGTGTGGTCATTATGgag                                           <  1:286380/288‑1 (MQ=255)`  
                                                                                                                                                                                                                                                              `ggATTCGTGCGTTACAGGATACTATTCGTGAACTGCAACGTAAGCAGGAAATCAAGTAAGGAGGCAATGTGT‑CTACTCAATCCAATCGTAATGCGCTCGTAGTGGCGCAACTGAAAGGAGACTTCGTGGCGTTCCTATTCGTCTTATGGAAGGCGCTAAACCTACCGGTGCCCACTAAGTGTCAGATTGACATGGCTAAGGTGCTGGCGAATGGAGACAACAAGAAGTTCATCTTACAGGCTTTCCGTGGTATCGGTAAGTCGTTCATCACATGTGCGTTCGTTGTGTGGTCCTTATGgag                                           <  1:67586/301‑1 (MQ=255)`  
                                                                                                                                                                                                                                                                `tttCGTGCTTTAAAGGATACTATTCTTGAACTGCAACGTAAGGAGGAAATCAAGTAAGGAGGCAATGTGT‑CTACTCAATCCAATCGTAATGCGCTCGTAGTGGCGCAACTGAAAGGAGACTTCGTGGCGTTCCTATTCGTCTTATGGAAGGCGCTAAACCTACCGGTGCCCACTAAGTGTCAGATTGACATGGCTAAGGTGCTGGCGAATGGAGACAACAAGAAGGTCATCTTACAGGCTTTCCGTGGTATCGGTAAGTCGTTCATCACATGTGCGTTCGTTGTGTGGTCCTTATGgagag                                         <  1:153559/300‑1 (MQ=255)`  
                                                                                                                                                                                                                                                                      `gCGTTACAGGATACTATTCGTGAACTGCAACGTAAGCAGGAAATCAAGTAAGGAGGCAATGTGT‑CTACTCAATCCAATCGTAATGCGCTCGTAGTGGCGCAACTGAAAGGAGACTTCGTGGCGTTCCTATTCGTCTTATGGAAGGCGCTAAACCTACCGTTGCCCACTAAGTGTCAGATTGACATGGCTAAGGTGCTGGCGAATGGAGACAACAAGAAGTTCATCTTACAGGCTTTCCGTGGTATCGGTAAGTCGTTCATCACATGTGCGTTCGTTGTGTGGTCCTTATGGAGAGACCCTc                                   >  1:208553/1‑301 (MQ=255)`  
                                                                                                                                                                                                                                                                           `aCAGGATACTATTCGTGAACTGCAACGTAAGCAGGAAATCAAGTAAGGAGGCAATGTGT‑CTACTCAATCCAATCGTAATGCGCTCGTAGTGGCGCAACTGAAAGGAGACTTCGTGGCGTTCCTATTCGTCTTATGGAAGGCGCTAAACCTACCGGTGCCCACTAAGTGTCAGATTGACATGGCTAAGGTGCTGGCGAATGGAGACAACAAGAAGTTCATCTTACTGGCTTTCCGTGGTATCGGTAAGTCGTTCATCACATTTGCGTTCGTTGTTTGGTCCTTATGGAGAGCCCCTcctttt                              >  1:225460/1‑296 (MQ=255)`  
                                                                                                                                                                                                                                                                           `aCAGGATACTATTCGTGAACTGCAACGTAAGCAGGAAATCAAGTAAGGAGGCAATGTGT‑CTACTCAATCCAATCGTAATGCGCTCGTAGTGGCGCAACTGAAAGGAGACTTCGTGGCGTTCCTATTCGTCTTATGGAAGGCGCTAAACCTACCGGTGCCCACTAAGTGTCAGATTGACATGGCTAAGGTGCTGGCGAATGGAGACAACAAGAAGTTCATCTTACAGGCTTTCCGTGGTATCGGTAAGTCGTTCATCACATGTGCGTTCGTTGTGTGGTCCTTATGGAGAGACCCTCAGTTg                              >  1:238798/1‑301 (MQ=255)`  
                                                                                                                                                                                                                                                                            `acagATACTATTCGTGAACTGCAACGTAAGCAGGAAATCAAGTAAGGAGGCAATGTGT‑CTACTCAATCCAATCGTAATGCGCTCGTAGTGGCGCAACTGAAAGGAGACTTCGTGGCGTTCCTATTCGTCTTATGGAAGGCGCTAAACCTACCGGTGCCCACTAAGTGTCAGATTGACATGGCTAAGGTGCTGGCGAATGGAGACAACAAGAAGTTCATCTTACAGGCTTTCCGTGGTATCGGTAAGTCGTTCATCACATGTGCGTTCGTTGTGTGGTCCTTATGGAGAGACCCTCAGTTGa                             <  1:119422/298‑1 (MQ=255)`  
                                                                                                                                                                                                                                                                             `aGGATACTATTCGTGAACTGCAACGTAAGCAGGAAATCAAGTAAGGAGGCAATGTGT‑CTACTCAATCCAATCGTAATGCGCTCGTAGTGGCGCAACTGAAAGGAGACTTCGTGGCGTTCCTATTCGTCTTATGGAAGGCGCTAAACCTACCGGTGCCCACTAAGTGTCAGATTGACATGGCTAAGGTGCTGGCGAATGTAGCCAACAAGAAGTTCATCTTACAGGCTTTCCGTGGTATCGGTAAGTCGTTCATAACATGTGCGTTCGTTGTGTGGTCCTTATgctgtctcttatacacatt                            >  1:155518/1‑283 (MQ=255)`  
                                                                                                                                                                                                                                                                              `ggATACTATTCGTGAACTGCAACGTAAGCAGGAAATCAAGTAAGGAGGCAATGTGT‑CTACTCAATCCAATCGTAATGCGCTCGTAGTGGCGCAACTGAAAGGAGACTTCGTGGCGTTCCTATTCGTCTTATGGAAGGCGCTAAACCTACCGGTGCCCACTAAGTGTCAGATTGACATGGCTAAGGTGCTGGCGAATGGAGACAACAAGAAGTTCATCTTACAGGCTTTCCGTGGTATCGGTAAGTCGTTCATCACATGTGCGTTCGTTGTGTGGTCCTTATGGAGAGACCCTCAGTTGAAg                           <  1:163275/301‑1 (MQ=255)`  
                                                                                                                                                                                                                                                                              `ggATACTATTCGTGAACTGCAACGTAAGCAGGAAATAAAGTAAGGAGGCAATGTGT‑CTACTCAATCCAATCGTAATGCGCTCGTAGTGGCGCAACTGAAAGGAGACTTCGTGGCGTTCCTATTCGTCTTATGGAAGGCGCTAAACCTACCGGTGCGCACTAAGTGTCAGATTGACATGGCTAAGGTGCTGGCGAATGGAGACAACAAGAAGTTCATCTTACAGGCTTTCCGTGGTATCGGTAAGTCGTTCATCACATGTGCGTTCGTTGAGCGGGCCTTATGGAGAGACCCTAAGTTGatg                           >  1:318112/1‑299 (MQ=255)`  
                                                                                                                                                                                                                                                                              `ggATACTATGAGTGAAATGAAACGTAAGCGAGAAATCAAGTAAAGAGGAAATGTGA‑CTACTAAATCCAAACGTAATGCGCTCGTAGTGGCGCAAATGAAAGGAGACTTCGTGGCGTTCCTATTCGTCTTATGGAAGGCGCTAAACTTACCGGTGCCCACTAAGTGTCAGATTGACATGGCTAAGGTGCTGGCGAATGGAGACAACAAGAAGTTCATCTTACAGGCTTTCCGTGGTATCGGTAAGTCGTTCATCACATGTGCGTTCGTTGTGTGGTCCTTATGGAGAGACCCTCAGTTGAAg                           <  1:227738/301‑1 (MQ=255)`  
                                                                                                                                                                                                                                                                                `aTACTATTCGTGAACTGCAACGTAAGCAGGAAATCAGGTAAGGAGTCAATGTGT‑CTACTCAATCCAATCGTAATGCGCTCGTAGTGGCGCAACTGAAAGGAGACTTCGTGGCGTTCCTATTCGTCTTATGGAAGGCGCTAAACCTACCGGTGCCCACTAAGTGTCAGATTGACATGGCTAAGGTGCTGGCGAATGGAGACAACAAGAAGTTCATCTTACAGGCTTTCCGTGGTATCGGTAAGTCGTTCATCACATGTGCGTTCGTTGTGTGGTCCTTATGGAGAGACCCTCAGTTGAAgct                         >  1:211018/1‑299 (MQ=255)`  
                                                                                                                                                                                                                                                                                `aTACTATTCGTGAACTGCAACGTAAGCAGGAAATCAAGTAAGGAGGCAATGTGT‑CTACTCAATCCAATCGTAATGCGCTCGTAGTGGCGCAACTGAAAGGAGACTTCGTGGCGTTCCTATTCGTCTTATGGAAGTCGCTAAACCTACCGGTGCCCACTAAGTGTCAGATTGACATGGCTACGGTGCTGGCGAATGGAGACAACAAGAAGTTCATCTTACAGGCTTTCCGTGGTATCGGTAAGTCGTTAATCACATGTTCGTTCGTTGTGTGGTCCTTATGGAGAGACCCTCCGTTGAAGAt                         >  1:231026/1‑301 (MQ=255)`  
                                                                                                                                                                                                                                                                                   `cTATTCGTGAACTGCAACGTAAGCAGGAAATCAAGTAAGGAGGCAATGTGT‑CTACTCAATCCAATCGTAATGCGCTCGTAGTGGCGCAACTGAAAGGAGACTTCGTGGCGTTCCTATTCGTCTTATGGAAGGCGCTAAACCTACCGGTGCCCACTAAGTGTCAGATTGACATGGCTAAGGTGCTGGCGAATGGAGACAACAAGAAGTTCATCTTACAGGCTTTCCGTGGTATCGGTAAGTCGTTCATCACATGTGCGTTCGTTGTGTGGTCCTTATGGAGAGACCCTCAGTTGAAGAtctg                      >  1:272399/1‑298 (MQ=255)`  
                                                                                                                                                                                                                                                                                    `tATTCGTGAACTGCAACGTAAGCAGGAAATCAAGTAAGGAGGCAATGTGT‑CTACTCAATCCAATCGTAATGCGCTCGTAGTGGCGCAACTGAAAGGAGACTTCGTGGCGTTCCTATTCGTCTTATGGAAGGCGCTAAACCTACCGGTGCCCACTAAGTGTCAGATTGACATGGCTAAGGTGCTGGCGAATGGAGACAACAAGAAGTTCATCTTACAGGCTTTCCGTGGTATCGGTAAGTCGTTCATCACATGTGCGTTCGTTGTGTGGTCCTTATGGAGAGACCCTCAGTTGAAGATACtt                     <  1:204920/301‑1 (MQ=255)`  
                                                                                                                                                                                                                                                                                                      `tAAGCAGGAAATCAAGTAAGGAGGCAATGTGT‑CTACTCAATCCAATCGTAATGCGCTCGTAGTGGCGCAACTGAAAGGAGACTTCGTGGCGTTCCTATTCGTCTTATGGAAGGCGCTAAACATACCGGTGCCCACTAAGTGTCAGATTGACATGGCTAAGGTGCTGGCGAATGGAGACAACAAGAAGTTCATCTTACAGGCTTTCCGTGGTATCGGTAAGTCGTTCATCACATGTGCGTTCGTTGTGTGGTCCTTATGGAGAGACCCTCAGTTGAAGATACTTATCGTATCAGCCTCTAAg   <  1:20171/301‑1 (MQ=255)`  
                                                                                                                                                                                                                                                                                                       `aaGCAGGAAATCAAGTAAGGATGCAAT‑TGTGCTACTAAATCCAATCGTAATGCGCTCGTAGTTGCGCCACTGAAAGGTGACTTCGTGGCGTTCCTATTCGTCTTATGGAAGGCGCTAAACCTACCGGTGCCCACTAAGTGTCAGATTGACATGGCTAAGGTGCTGGCGATTGGAGACAACAAGTGGTTCATCTTACAGGCTTTCCGTGGTATCGGTAAGTCGTTCATCACATGTGCGTTCGTTGTGTTGTCCTTATGGAGAGACCCTCAGTTGAAGATACTTATCGTATCAGCCTCTAAgg  <  1:40849/301‑1 (MQ=255)`  
                                                                                                                                                                                                                                                                                                       `aaGCAGGAAATCAAGTAAGGAGGCAATGTGT‑CTACTCAATCCAATCGTAATGCGCTCGTAGTGGCGCAACTGAAAGGAGACTTCGTGGCGTTCCTATTCGTCTTATGGAAGGCGCTAAACCTACCGGTGCCCACTAAGTGTCAGATTGACATGGCTAAGGTGCTGGCGAATGGAGACAACAAGAAGTTCATCTTACAGGCTTTCCGTGGTATCGGTAAGTCGTTCATCACATGTGCGTTCGTTGTGTGGTCCTTATGGAGAGACCCTCAGTTGAAGATACTTATCGTATCAGCCTCTAAgg  <  1:166555/301‑1 (MQ=255)`  
                                                                                                                                                                                                                                                                                                       `aaGCAGGAAATCAAGTAAGGAGGCAATGTGT‑CTACTCAATCCAATCGTAATGCGCTCGTAGTGGCGCAACTGAAAGGAGACTTCGTGGCGTTCCTATTCGTCTTATGGAAGGCGCTAAACCTACCGGTGCCCACTAAGTGTAAGATTGACATGGCTAAGGTGCTGGCGAATGGAGACAACAAGACGTTCATCTTACAGGCTTTCCGTGGTATCGGTAAGTCGTTCATCACATGTGCGTTCGTTGTGTGGTCCTTATGGAGAGACCCTCAGTTGAAGATACTTATCGTATCAGCCTCTAAgg  <  1:159538/301‑1 (MQ=255)`  

                                                                                                                                                                                                                                                                                                          `|`                                                                                                                                                                                                                                                                                                            
`AGTTGAGGCTGCGAAGAGCACTCAAAGAGCAATCGATGCGGTATCTGCTAAGTATCAAGAAGACCTTGCCGCGCTGGAAGGGAGCACTGATAGGATTATTTCTGATTTGCGTAGCGACAATAAGCGGTTGCGCGTCAGAGTCAAAACTACCGGAACCTCCGATGGTCAGTGTGGATTCGAGCCTGATGGTCGAGCCGAACTTGACGACCGAGATGCTAAACGTATTCTCGCAGTGACCCAGAAGGGTGACGCATGGATTCGTGCGTTACAGGATACTATTCGTGAACTGCAACGTAAGTAGGAAATCAAGTAAGGAGGCAATGTGT‑CTACTCAATCCAATCGTAATGCGCTCGTAGTGGCGCAACTGAAAGGAGACTTCGTGGCGTTCCTATTCGTCTTATGGAAGGCGCTAAACCTACCGGTGCCCACTAAGTGTCAGATTGACATGGCTAAGGTGCTGGCGAATGGAGACAACAAGAAGTTCATCTTACAGGCTTTCCGTGGTATCGGTAAGTCGTTCATCACATGTGCGTTCGTTGTGTGGTCCTTATGGAGAGACCCTCAGTTGAAGATACTTATCGTATCAGCCTCTAAGG  >  NC_001604.1/37048‑37643`

  

Alignment Legend

Aligned base mismatch/match (shaded by quality score): `ATCG/ATCG` `< 3 ≤` `ATCG/ATCG` `< 10 ≤` `ATCG/ATCG` `< 23 ≤` `ATCG/ATCG` `< 35 ≤` `ATCG/ATCG` `< 38 ≤` `ATCG/ATCG`

Unaligned base: `atcg`    Masked matching base: `atcg`    Alignment gap: `‑`    Deleted base: `‑`
